# Sulbactam–Durlobactam in the Treatment of Bloodstream Infection Due to Carbapenem-Resistant *Acinetobacter baumannii*: A Case Series and Literature Review

**DOI:** 10.3390/jcm15062281

**Published:** 2026-03-17

**Authors:** Ruiting Li, Limin Duan, Shengwen Sun, Dan Li, Qiwei Peng, You Shang

**Affiliations:** Department of Critical Care Medicine, Union Hospital, Tongji Medical College, Huazhong University of Science and Technology, Wuhan 430022, China; liminduan@hust.edu.cn (L.D.); 2020xh0076@hust.edu.cn (S.S.); lidanld@hust.edu.cn (D.L.); alexpeng@hust.edu.cn (Q.P.)

**Keywords:** *Acinetobacter baumannii*, carbapenem resistance, bloodstream infection, sulbactam–durlobactam, β-lactamase inhibitor

## Abstract

**Background:** Carbapenem-resistant *Acinetobacter baumannii* (CRAB), as a critical-priority pathogen among extensively drug-resistant (XDR) bacteria, influences healthcare-associated infections in China. Sulbactam–durlobactam (SUL-DUR) is recommended as a first-line agent for managing CRAB infections by the 2024 Infectious Diseases Society of America (IDSA) guidelines, but the usage of SUL-DUR for CRAB bloodstream infections (BSIs) has never been described in China. **Methods:** The present study aims to report five critically ill ICU patients recently treated in our center with CRAB BSIs who were successfully treated with SUL-DUR. Relevant literature and current guidelines are being briefly reviewed to provide a novel and promising clinical paradigm for managing BSI caused by CRAB. **Findings:** Use of SUL-DUR marked a definitive turning point in all five patients’ clinical trajectories. **Conclusions:** Our real-world evidence confirms SUL-DUR’s efficacy as a first-line agent for confirmed CRAB BSI cases and the definitive salvage therapy agent, impels a re-evaluation of the current clinical therapeutic spectrum for CRAB infection.

## 1. Introduction

*Acinetobacter baumannii* (AB) is a Gram-negative bacillus and a member of the *Acinetobacter calcoaceticus–A. baumannii* complex. As a prominent extensively drug-resistant (XDR) pathogen, it is frequently isolated from clinical specimens such as blood, urine, pus, and respiratory secretions in hospitalized patients [1]. AB develops resistance to most of the antibiotics through intrinsic and acquired mechanisms. Notably, Carbapenem-resistant *Acinetobacter baumannii* (CRAB) exhibits resistance to nearly all β-lactam antibiotics, including carbapenems, by producing class D β-lactamases (e.g., OXA-23, OXA-24/40, OXA-58), which hydrolyze these agents [2]. Among these, OXA-23 is the predominant carbapenemase genotype in CRAB, with a 99% detection rate in China [3]. In 2018, the World Health Organization (WHO) classified CRAB as a critical-priority pathogen among extensively drug-resistant (XDR) bacteria [4]. According to the 2024 CHINET data, CRAB constitutes approximately 70% of the total clinically isolated AB strains, underscoring its significant role in healthcare-associated infections and antimicrobial resistance in China [5].

A leading cause of severe sepsis and septic shock in intensive care unit (ICU) patients, bloodstream infection (BSI) is associated with higher morbidity, mortality, and treatment costs. BSIs occur when pathogens enter the bloodstream through invasive portals such as pre-existing infections, surgical wounds, intravenous catheters, or vascular devices. Subsequent hematogenous dissemination may lead to secondary infections and organ dysfunction [6]. A Chinese multicenter study (*n* = 2962 across 67 ICUs) identified Gram-negative bacilli as the predominant pathogens in ICU-acquired BSIs and revealed their association with independent drug resistance risk variables (OR = 2.18, 95%CI: 1.33–3.59) [7]. Another U.S. study confirmed AB is a common pathogen in ICU-acquired BSIs, with a notably higher prevalence of CRAB [8]. Particularly concerning are CRAB BSIs due to their poor prognosis—mortality rates exceed 60% [9], and their association with prolonged hospitalization and significantly increased healthcare costs, especially in ICUs [10].

Before the approval of sulbactam–durlobactam (SUL-DUR), the therapeutic options for CRAB infections were severely limited. Conventional regimens predominantly rely on polymyxin-based combination therapies. However, polymyxins demonstrate suboptimal efficacy against CRAB BSIs and are associated with a significant risk of nephrotoxicity [11]. According to the 2024 Infectious Diseases Society of America (IDSA) guidelines, SUL-DUR is recommended as a first-line agent for managing CRAB infections [12]. the alternative regimen for CRAB infection is high-dose ampilin–sulbactam in combination with polymyxin B, minocycinetigeccne, or ceiderocol [12]. Sulbactam, a β-lactam antibiotic, exhibits intrinsic activity against AB by inhibiting penicillin-binding proteins (PBPs), namely PBP1 and PBP3. Durlobactam is a broad-spectrum diazabicyclooctane (DBO) β-lactamase inhibitor that prevents sulbactam from hydrolysis by serine β-lactamases, including class A, C, and D enzymes. This synergistic combination effectively restores sulbactam’s activity against most of the CRAB strains. Furthermore, the phase III ATTACK trial demonstrated that SUL-DUR achieved favorable pharmacokinetic exposure and therapeutic plasma concentrations in patients with CRAB-associated hospital-acquired bacterial pneumonia (HABP) and ventilator-associated bacterial pneumonia (VABP), suggesting its potential clinical utility in treating BSIs [13].

The usage of SUL-DUR for CRAB BSIs has been documented in only three published cases to date [14,15,16]; however, none of them were reported from China, where the CRAB burden is substantial. Therefore, a critical unmet need for real-world clinical evidence regarding the efficacy and safety of SUL-DUR, especially among critically ill patients with BSIs. Here, we present the first case series from China, detailing five critically ill patients with CRAB BSIs who were successfully treated with SUL-DUR. Our findings may offer valuable clinical insights into its real-world use and support its role as a first-line therapy for CRAB BSI infections.

## 2. Case Series

### 2.1. Case 1

In September 2025, an 80-year-old female with a history of hypertension and cerebral infarction presented to the Emergency Department of Wuhan Union Hospital with abdominal pain, hematuria, and fever (39 °C) lasting for one day. She had initially been treated at a local hospital, and subsequent abdominal computerized tomography (CT) revealed urinary tract stones in the right renal calyx and right ureter. After admission to the Department of Critical Care Medicine (CCM), she developed septic shock, progressive acute respiratory distress syndrome (ARDS), thrombocytopenia, coagulation function abnormalities, acute kidney injury (AKI), and acute liver injury (ALI). Blood and sputum cultures were negative, but metagenomic next-generation sequencing (mNGS) of urine detected *Escherichia coli* (read count: 12 in number with a relative abundance of 0.07%). Following four days of empiric antibiotic therapy (meropenem + vancomycin), along with fluid resuscitation, endotracheal intubation, and lung-protective mechanical ventilation (tidal volume: 6–8 mL/kg, PEEP: 8–10 cmH_2_O), her shock condition improved significantly. On Day 3, sputum culture yielded CRAB with heavy (4+) semi-quantitative growth. Antibiotic susceptibility results are detailed in Table 1. Given the reduction in infection markers, significant improvement in lung imaging, and stable hemodynamics, CRAB was initially considered a colonizing bacterium. However, on Day 6, she experienced recurrent septic shock. Moreover, the mNGS report of bronchoalveolar lavage fluid (BALF) revealed AB (read count: 672,386, relative abundance of 99%), *Candida* spp. (read count: 245, relative abundance 92.3%). Meanwhile, urine mNGS detected several multiple organisms including *Proteus mirabilis* (read count: 4560, relative abundance: 99%), AB (read count: 210, relative abundance: 99%), *Escherichia coli* (read count: 104, relative abundance: 99%), *Nakaseomyces* (read count: 4454, relative abundance: 97.1%), and *Candida* spp. (read count: 46, relative abundance: 1.0%). Blood mNGS also identified AB (read count: 45, relative abundance: 99%). OXA-23 carbapenemase was subsequently confirmed in all AB isolates (Table 1). The initial rescue combination therapy with polymyxin B, tigecycline, and amphotericin B failed to control the progression of infection or reverse shock. On Day 8, her regimen was swithed to SUL-DUR plus imipenem-cilastatin, ceftazidime-avibactam, and amphotericin B. All repeat cultures (BALF, blood, and urine) were negative by Day 10. By that time, her hemodynamics was stable, and vasoactive agents were discontinued. Her oxygenation index was above 300 mmHg. Due to persistently elevated BALF 1,3-β-D-glucan levels, amphotericin B was continued. Owing to prolonged ventilator dependence, she underwent tracheostomy and received rehabilitation therapy. The patient’s clinical information is summarized in Table 2.

### 2.2. Case 2

In August 2025, a 69-year-old female was admitted to the Department of CCM at Wuhan Union Hospital due to progressive dyspnea lasting for one month. Pulmonary CT and laboratory tests confirmed the diagnosis of Sjögren’s syndrome-associated interstitial pneumonia. Initial management included glucocorticoids (for autoimmune lung disease), invasive mechanical ventilation (for respiratory failure), and broad-spectrum empirical antibiotics, such as ceftriaxone plus ganciclovir, for possible bacterial or viral coinfection. One week later, she developed septic shock following bronchoscopy. BALF culture revealed the presence of CRAB with heavy (3+) semi quantitative growth. Antibiotic susceptibility results are shown in Table 1. Additionally, mNGS of blood detected AB (OXA-23 carbapenemase-producing, read count: 764, relative abundance: 99%) and *Klebsiella pneumoniae* (read count: 21, relative abundance: 99%). BALF mNGS also confirmed AB (OXA-23 carbapenemase-producing, read count: 482,620, relative abundance: 99%) (Table 1). Given the family’s preference for aggressive treatment, she was administered SUL-DUR in combination with imipenem-cilastatin. Subsequently, her shock resolved and infection markers improved after sex days. However, due to the higher cost of SUL-DUR, therapy was switched to polymyxin B plus tigecycline. Three days later, the patient developed recurrent fever along with elevated white blood cell (WBC) and procalcitonin (PCT) levels. Consequently, SUL-DUR and imipenem-cilastatin were reintroduced. After ten days of treatment, her WBC and PCT levels normalized, and blood cultures were negative. Subsequent sputum culture identified *Stenotrophomonas maltophilia*, leading to treatment with levofloxacin and minocycline. Despite immunosuppressive treatment (glucocorticoids, immunoglobulin, and rituximab), her interstitial pneumonia progressed, resulting in refractory hypoxemia and death. The clinical information of the patient is summarized in Table 2.

### 2.3. Case 3

In August 2025, a 34-year-old male presented to the Emergency Department with chest pain lasting for nine hours. Aortic computed tomography angiography (CTA) performed at the local hospital revealed type A aortic dissection, and he was scheduled for hospital admission and surgery. Three days later, due to the progression of respiratory failure, he was admitted to the Department of Critical Care Medicine (CCM) for endotracheal intubation and mechanical ventilation. Consequently, the surgery was postponed. The diagnosis was type A aortic dissection complicated by ARDS and pulmonary infection. Based on the sputum culture’s results indicating *Staphylococcus aureus* and AB, the initial anti-infection regimen consisted of linezolid and cefuroxime. During this period, his fever and respiratory failure improved significantly. Nine days after admission to the Department of CCM, he developed a recurrent fever (39.2 °C), accompanied by markedly enhanced PCT and WBC levels. As the sputum culture revealed CRAB with heavy (3+) semi quantitative growth (Table 1), anti-infective therapy was adjusted to tigecycline and imipenem-cilastatin. However, his fever persisted at 40 °C, and the PCT level rose to 7.5 ng/mL. Subsequent mNGS of blood detected AB (producing OXA-23 carbapenemase, read count: 303, relative abundance: 99%) (Table 1) and Hepatitis B virus (read count: 3536, relative abundance: 99%). Treatment was then switched to SUL-DUR combined with imipenem-cilastatin, along with entecavir. The patient’s peak body temperature and PCT levels decreased significantly. After one week, when his vital signs had stabilized and laboratory results were nearly normal, he underwent aortic dissection surgery. The clinical information of the patient is summarized in Table 2.

### 2.4. Case 4

A 33-year-old man with a history of intestinal obstruction and Meckel’s diverticulum presented with abdominal pain and fever for over a month. In August 2025, he was transferred from a local hospital to the Department of CCM of Wuhan Union Hospital. A CT scan from the referring hospital had shown incomplete intestinal obstruction and pulmonary infection, with a maximum recorded body temperature of 38.5 °C. Twenty days prior to transfer, he underwent endotracheal intubation and mechanical ventilation due to type I respiratory failure. Sputum culture one day after intubation revealed CRAB (Table 1). Due to failure to wean from the ventilator, a tracheotomy was performed ten days before transfer. Upon admission to the Department of CCM of Wuhan Union Hospital, the patient was in septic shock. Based on the outside hospital’s microbiological findings (CRAB, Table 1), combination anti-infection therapy with polymyxin B, tigecycline, and daptomycin was initiated, along with fluid resuscitation and correction of metabolic acidosis. By the fifth hospital day septic shock persisted. Repeated PCT and WBC levels remained elevated. Meanwhile, mNGS results of blood confirmed CRAB (read count: 563, relative abundance: 99%) (Table 1). The anti-infection regimen was subsequently changed to high-dose sulbactam (>6 g/day), polymyxin B, and eravacycline. Due to persistently low oxygenation indices, prone positioning was also implemented. After five days of this regimen, CRP level had decreased, but PCT levels remained largely unchanged and WBC counts continued to rise. Given the lack of adequate infection control, antibiotics were adjusted to sulbactam–durlobactam (SUL–DUR) plus imipenem–cilastatin. Intermittent prone position ventilation was continued to improve oxygenation. Subsequently, in light of detected carbapenem-resistant *Pseudomonas aeruginosa* (CRPA), ceftazidime-avibactam was added. During this period, due to fluctuating fever and inflammatory markers, BALF NGS was performed and again indicated CRAB (read count: 5437, relative abundance:99%) and CRPA (read count: 3782, relative abundanc: 99%). Due to concern for New Delhi metallo-β-lactamase (NDM)-producing CRPA, aztreonam was further added to the regimen. After 11 days of SUL-DUR-based anti-infection therapy, the infection was controlled. The patient was eventually discharged to a rehabilitation hospital for further management. Clinical information is summarized in Table 2.

### 2.5. Case 5

A 71-year-old woman with a history of coronary heart disease experienced drowning and was initially treated at a local hospital. She presented with breathing difficulties and impaired consciousness. As her consciousness disorder worsened and she developed a fever of 41 °C, she was transferred to the Department of CCM at Wuhan Union Hospital for further treatment. A CT scan from the local hospital revealed lacunar infarction and brain atrophy. It was considered that the impaired consciousness and high fever were related to sepsis and pulmonary infection. Additionally, she developed acute coronary syndrome after admission, manifested by a significantly elevated high-sensitive Troponin I level and ST-T changes on the electrocardiogram. Based on empirical anti-infection therapy (piperacillin-tazobactam plus levofloxacin), clopidogrel and atorvastatin were also administered. After five days, her infection and impaired consciousness disorder improved, and she was transferred to the Internal Medicine department for a coronary angiogram. Three days later, she was transferred back to the Department of CCM due to the onset of septic shock. Empirical treatment with cefperazone-sulbactam was then initiated. Since CRAB was detected in the blood culture results (Table 1) and *Candida krusei* was found in the urine, an anti-infective treatment with SUL-DUR, minocycline, and amphotericin B was started. After six days of treatment, her septic shock improved significantly. Repeat blood culture results were negative. She was then transferred back to the Internal Medicine Department. Finally, after another ten days of treatment in the Internal Medicine Department and undergoing percutaneous coronary intervention, she was discharged. Clinical information is summarized in Table 2.

## 3. Literature Review

### 3.1. Challenges in Treating CRAB

CRAB infections are challenging in critical care settings, where ICU mortality rates can cross 40–70%. This poor prognosis is significantly attributed to the pathogen’s multifaceted resistance phenotype, thereby leading to a therapeutic dead end and severely limiting the available antimicrobial therapies [17,18].

Colistin is a long-standing cornerstone in treating such cases. However, its effectiveness is hampered by a serious pharmacodynamic paradox. Although the in vitro resistance rate is low (4%; 95% CI, 3–5) [19], the clinical mortality rate among patients on colistin consistently surpasses 40%. This striking disparity questions the reliability of in vitro susceptibility as an indicator of clinical efficacy. However, this therapeutic shortfall is exacerbated by significant safety concerns. Since the incidence of AKI is approximately 36.2% [20], its poor lung penetration limits its use in pneumonia cases [21]. Although there are options for nebulization regimens as a supplement to the drug concentration in the alveolar epithelial lining fluid, no obvious clinical benefits have been observed in current studies.

Tetracycline derivatives are also ineffective as a viable alternative because of a critical pharmacokinetic/pharmacodynamic (PK/PD) mismatch. Despite showing a potent in vitro activity [minimum inhibitory concentration (MIC) 90 ≤ 2 mg/L], tigecycline achieves sub-therapeutic plasma concentrations. Consequently, it is usually ineffective for treating systemic infections [22,23,24]. In fact, MIC values of ≥2 μg/mL exhibit a significantly increased mortality rate, thereby questioning the clinical relevance of its breakpoints [25,26,27,28]. The newer analogue, eravacycline, has failed to deliver either, with one study reporting that the 30-day mortality rate was higher in eravacycline cases, compared to colistin-based regimens (33% vs. 15%; *p* = 0.048) [29]. These findings are in line with the 2024 IDSA guidelines, which forbid tetracycline derivatives in treating BSIs [12].

Similarly, the clinical relevance of sulbactam has been significantly undermined by widespread resistance. Having intrinsic activity, the non-susceptibility rates of sulbactam in regions, such as China, now approach 90%, while the susceptibility rate rarely exceeds 40% worldwide [30]. Moreover, over half of Chinese isolates already showed high-level resistance (MIC ≥ 16 mg/L) more than a decade ago [31]. Hence, empirical high-dose strategies are clinically unjustifiable in such cases.

Cefiderocol, a novel Siderophore Cephalosporin, which possesses a broad activity against CRAB, has been approved by the FDA for the treatment of infections caused by CRAB [32]. In 2024, IDSA guidelines suggested that an alternative regimen for CRAB infection is high-dose ampilin–sulbactam in combination with polymyxinB, minocycinetigeccne, or ceiderocol [12]. But the current evidence suggests that this combination of other antibiotics compared to ceiderocol does not improve clinical outcomes of patients infected with CRAB [33].

In summary, the therapeutic scenario for CRAB infections exhibits systemic ineffectiveness. Our first-line agents display high toxicity, suboptimal pharmacokinetics, and a significant disparity between in vitro activity and clinical efficacy. All these parameters are exacerbated by the increasing resistance. Hence, novel, targeted therapies are now necessary to manage these well-defined challenges.

### 3.2. SUL-DUR: A Novel Therapeutic Option

CRAB-associated infections are a serious public concern due to the increasing prevalence of Gram-positive bacteria and their antibiotic resistance. SUL-DUR is now a pivotal first-line treatment for CRAB-associated infections. Its synergistic mechanism involves inhibiting Class A, C, and D β-lactamases through its broad-spectrum β-durlobactam inhibition, which additionally enhances sulbactam’s intrinsic bactericidal activity. This synergy effectively shields it from these enzymatic interferences. As per the ATTACK trial, SUL-DUR has been significantly efficacious, relative to colistin-based therapy. The reduction in all-cause mortality from 32.3% to 19.0% underscores its superior efficacy, it also achieves a significantly higher clinical cure rate of 61.9% (vs. 40.3%) [13]. This efficacy is crucial for endemic regions, like China, where over 97.9% of isolates remain susceptible despite an enhanced imipenem resistance (84.6%) [30]. Crucially, this enhanced efficacy is coupled with a superior safety profile, effectively widening its therapeutic index. The ATTACK trial also revealed a lower incidence of serious adverse events in the SUL-DUR arm versus the colistin-based therapy (12.7% vs. 20.9%), with no drug-attributable mortality [13]. Collectively, both the superior efficacy and enhanced safety of SUL-DUR establish it as a new standard of care for CRAB-associated infections. This position is now codified by the 2024 IDSA guidelines, which recommend using it as a first-line treatment, particularly in cases of colistin resistance or intolerance [12].

### 3.3. Challenges in Treating CRAB-BSIs

CRAB- BSIs are a clinical challenge, with 30-day mortality rates crossing 50–70%. We posit that this therapeutic failure in BSIs results from pharmacokinetic limitations. i.e., the conventional agents’ inability to achieve sustained, bactericidal plasma exposures. Legacy agents exhibit several significant pharmacological shortcomings. Tigecycline’s tissue sequestration causes subtherapeutic plasma concentrations (<1 mg/L), thereby prompting an FDA black box warning for increased all-cause mortality [34]. Similarly, colistin’s efficacy is limited by a narrow therapeutic index, characterized by high nephrotoxicity and unpredictable pharmacokinetics. Thus, these limitations have created a critical therapeutic gap for CRAB BSI patients [35,36]. Consequently, SUL-DUR has emerged as a pharmacologically optimized solution to achieve robust plasma exposures. Its administration yields peak concentrations >100 mg/L and >50 mg/L for sulbactam and durlobactam, ensuring a >90% probability of achieving bactericidal pharmacodynamic targets against CRAB (MIC ≤ 4 mg/L), respectively. This optimized PK/PD profile matched to favorable outcomes in the BSI subgroup (n = 21) of the pivotal ATTACK trial. Additional real-world evidence supports these findings [13]. Mangioni et al. [14] reported the resolution of a CRAB bacteremia resistant to a tigecycline-containing regimen, demonstrating SUL-DUR’s efficacy after prior therapeutic failure. Similarly, VanNatta et al. [15] documented its successful usage in a critical burn patient, highlighting its clinical utility in complex cases.

### 3.4. SUL-DUR Coinfection and New Target

We explored SUL-DUR’s efficacy in monomicrobial CRAB infections to clarify its strategic role within the arena of critical care infections. We first used SUL-DUR for CRAB-BSIs, where IDSA guidelines strongly recommend a combination therapy. Our analysis identified a compelling synergistic effect between SUL-DUR and imipenem. In this scenario, durlobactam provides dual protection against OXA-type enzymes while the sulbactam exhibits complementary PBP binding [37,38]. Hence, this synergy provides a rational framework for maximizing bactericidal potency and suppressing resistance.

The clinical imperative for this strategy is underscored by an enhanced prevalence of polymicrobial infections. Furthermore, ATTACK trial data reveal that nearly one-third of patients with CRAB infections presented with co-pathogens, like other MDR organisms, like *Klebsiella pneumoniae* (77% non-susceptible to imipenem) and *Pseudomonas aeruginosa* (58% non-susceptible to imipenem). In this cohort, the SUL-DUR regimen yielded favorable outcomes than colistin in 28-day mortality (22% vs. 26%) and clinical cure (59.3% vs. 56.2%) [13]. Consequently, we interpret this as a clinical utility of SUL-DUR against the pathogen landscape of these infections.

Additionally, preclinical data on SUL-DUR’s extended spectrum provide the pharmacological basis. Furthermore, the antimicrobial spectrum of SUL-DUR is not limited to AB. Many in vitro and in vivo studies demonstrate its activity against a range of other crucial Gram-negative bacteria. For instance, it is potent against Enterobacterales (MIC 0.5–2 µg/mL), including some NDM-producing isolates [39,40]. However, it exerts synergistic effects against resistant *Pseudomonas aeruginosa* and *Enterobacterales* when combined with cefepime or carbapenems, thereby restoring their susceptibility [41,42]. Additionally, it has also shown potential against the rare but challenging *Burkholderia* species [43].

Therefore, our integrated analysis suggests that the therapeutic paradigm for SUL-DUR should evolve beyond niche monotherapy. Its primary value may be derived from its capacity as a cornerstone agent for severe, mixed Gram-negative infections. Hence, future clinical research should focus on elucidating optimal partner agents, defining SUL-DUR’s role in empirical regimens for suspected MDR infections, and assessing its clinical impact on both patient survival and the antimicrobial resistance ecosystem.

## 4. Discussion

Our description of these five critically ill patients illuminates the profound therapeutic futility of conventional antimicrobial strategies against infections caused by OXA-23-producing CRAB. We also observed a consistent pattern of clinical failures, where using last-resort agents, including polymyxin B and tigecycline, failed to achieve infection control and led to refractory septic shock. This reality underscores a critical challenge in the spectrum of contemporary medicine: a therapeutic armamentarium severely depleted by widespread resistance. This challenge is compounded by the available agents’ inherent pharmacological liabilities. The known nephrotoxicity of polymyxin B and the suboptimal serum concentrations achieved by tigecycline—a fatal therapeutic fault for bacteremia—represent key mechanistic factors behind our cohort’s treatment failures.

In this context, using mNGS in our diagnostic workflow proved indispensable for breaking the therapeutic deadlock. For instance, mNGS decisively resolved the clinical ambiguity of colonization versus infection in Case 1. The overwhelming microbial burden, quantified by an enhanced read count (672,386) and relative abundance (99%) in the BALF, implicated CRAB as the etiological agent of pneumonia and mandated an immediate therapeutic escalation. In Cases 3 and 4, the rapid molecular identification of both the pathogen and its OXA-23 resistance determinant from blood samples provided relevant data before conventional culture reports. This crucial diagnostic acceleration enabled us to avoid prolonged ineffective empirical therapy and promptly initiate a targeted SUL-DUR regimen.

Use of SUL-DUR marked a definitive turning point in all five patients’ clinical trajectories. We documented a rapid and consistent clinical response—characterized by hemodynamic stabilization and reduced PCT levels—within three to six days of administration. The key in vivo demonstration of its efficacy is presented in Case 2, where the patient’s relapse post-SUL-DUR withdrawal and subsequent recovery after its re-initiation serves as a crucial intra-patient control, thereby validating its potent antimicrobial activity. We attribute this superior clinical performance primarily to SUL-DUR’s advantageous PK/PD profile. SUL-DUR can achieve and sustain plasma concentrations well above the minimum inhibitory concentration required for bactericidal effects against CRAB. This PK superiority is helpful for treating BSIs and is responsible for achieving significant bloodstream clearance, as verified by microbiological eradication in Case 5.

Our experience indicates that the optimal implementation of SUL-DUR can become the cornerstone of a broader, individualized combination regimen. The frequent co-administration of SUL-DUR with imipenem-cilastatin (Cases 2, 3, and 4) aligns with the established pharmacological principles that aim at achieving synergy and suppressing resistance. This strategic approach moves beyond monotherapy to construct an efficacious therapeutic defense. Our management of Case 4 exemplifies this dynamic, etiology-driven strategy. After identification of CRPA, this regimen was expanded to include ceftazidime-avibactam and aztreonam. This underscores the principle that a SUL-DUR-based platform facilitates tailored, multi-agent strategies that are responsive to the entire microbiological landscape for critically ill patients with polymicrobial or evolving infections.

## 5. Conclusions

In conclusion, our case series delineates a novel and promising clinical paradigm for managing life-threatening BSI caused by OXA-23-producing CRAB. This paradigm integrates the diagnostic precision of mNGS with SUL-DUR’s therapeutic potency. This combination creates a diagnosis-to-treatment continuum that effectively circumvents the delays in traditional microbiology and overcomes the PK/PD deficiencies of conventional last-resort agents. Our real-world evidence confirms SUL-DUR’s efficacy as a salvage therapy and impels a re-evaluation of the current clinical therapeutic spectrum for CRAB infection. These compelling outcomes underscore the need for prospective, randomized controlled trials (RCTs). The objective of such trials should be to codify SUL-DUR’s role within treatment algorithms and determine its optimal utility—whether as a first-line agent for confirmed CRAB BSI cases or as the definitive salvage therapy agent. Thus, widespread implementation of this strategy could represent a pivotal advancement in our therapeutic armamentarium against XDR pathogens.

## Figures and Tables

**Table 1:** Results of antibiotic susceptibility and antibiotic resistance genes.

Positive *A. baumannii* Culture		MIC (mg/L)							Carbapenemase
Patient	Source	MC	MEM	IMP	CST	ERV	SUL-DUR	SMZ	
Case 1	sputum	13 I	>8 R	>8 R	2 I		22 S	>2/38 R	OXA-23
	blood								OXA-23
Case 2	BALF	14 I	>8 R	>8 R	1 S		21 S	>2/38 R	OXA-23
	blood								OXA-23
Case 3	sputum	11 S	>8 R	>8 R	2 I	16 S		>2/38 R	
	blood								OXA-23
Case 4	sputum	13 I	>8 R	>8 R	2 I	15 S	23 S	<0.5/9.5 S	
	blood								OXA-23
Case 5	blood	13 I	>8 R	>8 R	2 I		23 S	>2/38 R	
	blood	13 I	>8 R	>8 R	2 I		23 S	>2/38 R	

MIC: minimum inhibitory concentration; MC: minocycline; MEM: meropenem; IMP: imipenem; CST: colistin; ERV: eravacycline; SMZ: compound Sulfamethoxazole; BALF: bronchoalveolar lavage fluid; SUL-DUR: sulbactam–durlobactam; I: intermediate; R: resistant; S: susceptible.

**Table 2:** Clinical information of patients.

Patient	Age (Years)	Gender	Comorbidity	Laboratory Parameters During CRAB Infection	Maximum Body Temperature (°C)	Infection Site	Microbiological Data (mNGS)	Antibiotic Treatment Plan During CRAB Infection	Treatment Duration of SUL-DUR (Days)
Case 1	80	female	hypertension, cerebral infarction	CRP: 266 mg/L WBC: 23.32 × 10^9^ /L	39	urinary system, lung, blood	BALF: CRAB, *Candida* spp.; Urine: *Proteus mirabilis*, CRAB, *Escherichia coli*, *Nakaseomyces*, and *Candida* spp. Blood: CRAB.	SUL-DUR, imipenem-cilastatin, ceftazidime-avibactam, amphotericin B	7
Case 2	69	female	Sjögren’s syndrome	PCT: 7.5 ng/mL CRP: 247 mg/L WBC: 26.42 × 10^9^ /L	38.7	lung, blood	BALF: CRAB; Blood: CRAB, *Klebsiella pneumoniae*.	SUL-DUR, imipenem-cilastatin	6
Case 3	34	male	type A aortic dissection	PCT: 7.5 ng/mL CRP: 243 mg/L WBC: 17.22 × 10^9^ /L	40	lung, blood	Blood: CRAB, hepatitis B virus.	SUL-DUR, imipenem-cilastatin, entecavir	7
Case 4	33	male	intestinal obstruction, Meckel’s diverticulum	PCT: 11.0 ng/mL CRP: 209 mg/L WBC: 16.05 × 10^9^ /L	38.8	lung, blood	Blood: CRAB; BALF: CRAB, CRPA.	SUL-DUR, imipenem-cilastatin, aztreonam, ceftazidime-avibactam	11
Case 5	71	female	coronary heart disease	CRP: 189 mg/L WBC: 17.27 × 10^9^ /L	41	urinary system, lung, blood	Blood: CRAB; Urine: *Candida krusei*.	SUL-DUR, minocycline, amphotericin B	6

SUL-DUR: sulbactam–durlobactam; BALF: bronchoalveolar lavage fluid; CRPA: carbapenem-resistant *Pseudomonas aeruginosa*; CRAB: *Acinetobacter baumannii*; mNGS: metagenomic next-generation sequencing; CRP: C-reaction protein; PCT: procalcitonin; WBC: white blood cell count.

## Data Availability

Data sharing is not applicable to this article.

## Abbreviations

AB	*Acinetobacter baumannii*
XDR	extensively drug-resistant
CRAB	Carbapenem-resistant *Acinetobacter baumannii*
WHO	World Health Organization
ICU	intensive care unit
BSI	bloodstream infection
SUL-DUR	sulbactam–durlobactam
IDSA	Infectious Diseases Society of America
PBPs	penicillin-binding proteins
DBO	diazabicyclooctane
HABP	hospital-acquired bacterial pneumonia
VABP	ventilator-associated bacterial pneumonia
CT	computerized tomography
CCM	Critical Care Medicine
ARDS	acute respiratory distress syndrome
AKI	acute kidney injury
ALI	acute liver injury
mNGS	metagenomic next-generation sequencing
BALF	bronchoalveolar lavage fluid
WBC	white blood cell
PCT	procalcitonin
CTA	computed tomography angiography
CRPA	*Pseudomonas aeruginosa*
PK/PD	pharmacokinetic/pharmacodynamic
MIC	minimum inhibitory concentration
RCTs	randomized controlled trials
